# The Trap Architecture of *Utricularia multifida* and *Utricularia westonii* (subg. *Polypompholyx*)

**DOI:** 10.3389/fpls.2019.00336

**Published:** 2019-03-26

**Authors:** Bartosz J. Płachno, Piotr Świątek, Lubomír Adamec, Samanta Carvalho, Vitor F. O. Miranda

**Affiliations:** ^1^Department of Plant Cytology and Embryology, Institute of Botany, Jagiellonian University, Kraków, Poland; ^2^Department of Animal Histology and Embryology, University of Silesia in Katowice, Katowice, Poland; ^3^Institute of Botany of the Czech Academy of Sciences, Třeboň, Czechia; ^4^Departamento de Biologia Aplicada à Agropecuária, Faculdade de Ciências Agrárias e Veterinárias, UNESP – São Paulo State University, São Paulo, Brazil

**Keywords:** Australian plant species, carnivorous plants, Lentibulariaceae, *Polypompholyx*, trap function, transfer cells, ultrastructure

## Abstract

*Utricularia* are carnivorous plants which have small hollow vesicles as suction traps that work underwater by means of negative pressure and watertightness of the entrance for capturing small animal prey. *Utricularia multifida* and *U. westonii* have specific thick-walled traps, which are triangular in a transverse section but their functioning is unclear. Some authors suggest that the trap door in *U. multifida* acts as a simple valve without a suction trapping mechanism. Our main aim was to check the anatomical trap characters that are responsible for possible water outflow and maintaining negative pressure as main functional parts of the active trap suction mechanism in both species. Using different microscopic techniques, we investigated the ultrastructure of external trap glands, quadrifids, glands near the entrance (bifids, monofids), and also pavement epithelium. Quadrifids of both species have a similar structure to those known in other species from the genus, which possess the suction trap mechanism. Glands near the entrance in *U. multifida* and *U. westonii*, which are responsible for water pumping in other species, are typically developed as in other species in the genus and have pedestal cells which are transfer cells. The transfer cells also occur in glands of the pavement epithelium, which is again typically developed as in other species in the genus. Simple biophysical tests did not confirm reliably neither the negative underpressure formation in the traps nor the watertightness of the entrance in both species. Our anatomical results indirectly support the hypothesis that both species have suction traps like all other *Utricularia* species, but the biophysical data rather suggest a passive valve mechanism.

## Introduction

The carnivorous genus *Utricularia* L. (Lentibulariaceae) contains around 240 species which are terrestrial, aquatic (or amphibious) or epiphytic (Taylor, 1989; Jobson et al., 2018). The plants are able to capture fine animal prey by their foliar traps. The traps in the most species are discoid, hollow bladders, usually 1–5 mm large with a typical trap wall thickness of two cells and they are filled with trap fluid (Lloyd, 1942; Taylor, 1989; Płachno et al., 2012; Westermeier et al., 2017). They contain a variety of glands and trichomes on both the inner and outer surfaces. The inner trap surface is covered by many large quadrifid glands (trichomes), the main function of which is the secretion of digestive enzymes, while the bifid glands situated close to the trap door take part in water pumping out of the trap and in formation of a negative pressure necessary for rapid trap firing (Lloyd, 1942; Fineran, 1985; Taylor, 1989; Westermeier et al., 2017). According to Fineran (1985), small sessile glands on the external trap wall are also responsible for secreting water from the trap to the environment. Thus, various glands could be responsible for proper *Utricularia* trap activity and water removal from the trap lumen to the external environment.

In the past, *Polypompholyx* Lehm. [*Polypompholyx multifida* (R.Br.) F.Muell. and *Polypompholyx tenella* (R.Br.) Lehm.] was treated as a genus separated from the genus *Utricularia* because of its unique trap morphology and four calyx lobes (e.g., Kamieński, 1891; Lang, 1901; Lloyd, 1932, 1936, 1942). Taylor (1989), in his outstanding monograph of the genus *Utricularia*, ranked *Polypompholyx* as a subgenus of *Utricularia* with two sections: *Polypompholyx* (Lehm.) P. Taylor (two species: *U. multifida* R. Br. and *U. tenella*) and *Tridentaria* P. Taylor (one species *U. westonii* P. Taylor). After the most recent taxonomical proposal, the subgenus *Polypompholyx* includes four sections: *Polypompholyx*, *Tridentaria*, *Pleiochasia* Kamieński and *Lasiocaules* R. W. Jobson, Reut & Baleeiro (Jobson et al., 2017, 2018).

Lloyd (1932, 1942) found traps of *U. multifida* and *U. tenella* “extremely curious” and mentioned their quite special characters:

(1) Special shape; traps are triangular in transverse section. The top part of the traps is almost flat. (2) The entrance to the traps has a complicated structure; there is a double reception chamber. The entrance to the traps from the front is blocked, so an access is only from lateral sides. (3) Histologically, the door is unique in the case of the “very great depth of the inner course cells in the upper hinge region” (Lloyd, 1942, p. 263). There is no obvious middle region (piece) of the door. “The door has quite a different form from that in *Utricularia*, being thickest and least flexible at the base....” (Lloyd, 1932, p. 323). (4) The entrance into the trap is very small relatively to the size of the trap. (5) Massive walls of trap (four layers of cells). (6) There are some differences in trichomatous armature of the threshold between large and small trap types.

At the beginning of his studies, Lloyd used only fixed material and mentioned some additional trap characters or lack thereof. In his first work (Lloyd, 1932), he noted e.g., that:

(1) There is no hinge mechanism, (2) the pavement epithelium displays no specialized regions and (3) there is no velum.

Lloyd proposed that in both species: “the door acts as a simple valve and is incapable of contributing to the sustention of a low pressure of water within the trap.” However, he also noted that “Whether the walls of the trap act as they do in *Utricularia*, producing a low pressure of water in the interior, or not, we cannot, in the absence of the study of living material by experimental methods, say; nor indeed can we be certain that the foregoing interpretation is correct. We can at the moment go only so far as the structural evidence seems to indicate.” (Lloyd, 1932). After studying living material, he corrected earlier observations (Lloyd, 1936, 1942) and found that *U. multifida* had active traps: “it was difficult to study the trap in action, and especially to photograph it. Nevertheless, the attempt succeeded.” (Lloyd, 1942, p. 257, see also his Plate 24, Figure 8). Using living material, he also observed velum (which was produced by glands from the outer zone of pavement epithelium) and interpreted short, bent glandular hairs as the tripping mechanism.

Reifenrath et al. (2006) suggested that in traps of *U. multifida*, a suction mechanism does not function but tunnel-shaped entrances leads to a door-less digesting chamber (similar to *Genlisea* traps). Unfortunately, these authors did not discuss Lloyd’s results in the case of the trap action of *U. multifida*. Probably, Lloyd’s observations did not fit into their hypothesis. Recently, Westermeier et al. (2017) investigated trap biomechanics in 19 *Utricularia* species including *U. multifida*. These authors observed neither suction action, trapdoor movements nor spontaneous firings of *U. multifida* traps. Thus, they proposed the *U. multifida* trap type to be passive. Similar to Reifenrath et al. (2006); Westermeier et al. (2017) proposed that *U. multifida* traps work in a passive manner similar to traps of the closely-related *Genlisea* and did not discuss Lloyd’s successful experiment on the trap activity of *U. multifida* either. On the other hand, Lönnig (2018) was critical and found some weak points in the argumentation of both of the last studies.

*Utricularia westonii* has been even more poorly studied than *U. multifida* and *U. tenella*. Taylor (1989) described the general trap morphology and the shape of internal trap glands of *U. westonii*. Accordingly, this species is unique in the whole subgenus *Polypompholyx*, due to the occurrence of tripping door bristles, which are lacking in species from other sections of this subgenus. The presence of tripping door bristles might suggest that *U. westonii* traps work in an active manner, because *Utricularia* species which have tripping door bristles typically also have a suction mechanism and an active trap. However, the trap wall is thick and rigid and consists of four cell layers (Płachno et al., 2015).

Our main aim was to study trap characters of *Utricularia multifida* and *U. westonii* which are responsible for a possible water outflow and can mirror trap functioning (passive functioning versus active suction mechanism). We focused on the ultrastructure of external trap glands, quadrifids, glands near the entrance and also of the pavement epithelium; structures which are considered to be involved in the water outflow from *Utricularia* traps (Fineran, 1985; Heide-Jørgensen, 1989). We also directly tested the possibility of trap firing.

## Materials and Methods

### Plant Material

Plant material of *U. multifida* (Walpole, SW, Australia) and *U. westonii* was obtained from the collection of Kamil Pásek (Ostrava, Czechia^1^) from an *in-vitro* culture. The plants were grown in a half-strength MS medium on gerlite gel at 19 ± 1°C in fluorescent light with a 14 h photoperiod. For each species, at least 20 adult traps 1.2–2 mm large were examined for anatomical studies.

### Methods

Traps were examined using light microscopy (LM), scanning electron microscopy (SEM), and transmission electron microscopy (TEM) as follows. Freshly excised traps were fixed in a mixture of 2.5 or 5% glutaraldehyde with 2.5% formaldehyde in a 0.05 M cacodylate buffer (Sigma; pH 7.2) overnight or for several days, washed three times in a 0.1 M sodium cacodylate buffer and post-fixed in a 1% osmium tetroxide solution at room temperature for 1.5 h. Dehydration using a graded ethanol series, infiltration and embedding using an epoxy embedding medium kit (Fluka) followed. Following polymerization at 60°C, sections for TEM were cut at 70 nm using a Leica ultracut UCT ultramicrotome, stained with uranyl acetate and lead citrate (Reynolds, 1963) and examined using a Hitachi H500 transmission electron microscope (Hitachi, Tokyo, Japan) at an accelerating voltage of 75 kV.

Semi-thin sections (0.9–1.0 μm thick) prepared for LM were stained for general histology using aqueous methylene blue/azure II (MB/AII) for 1–2 min (Humphrey and Pittman, 1974) and examined with an Olympus BX60 light microscope. The periodic acid-Schiff (PAS) reaction for LM (semi-thin sections) was also used to reveal the presence of insoluble polysaccharides, and Sudan Black B was used to detect the presence of lipids and cuticle material (Jensen, 1962). Staining for total proteins was performed using mercuric bromophenol blue (Mazia et al., 1953). For SEM, traps were fixed (as above) and later dehydrated and subjected to critical-point drying using CO_2_. They were then sputter-coated with gold and examined at an accelerating voltage of 20 kV using a Hitachi S-4700 scanning electron microscope, which is housed in the Institute of Geological Sciences, Jagiellonian University in Kraków, Poland. Living, non-fixed traps, were cut with a razor blade and examined with an Olympus BX60 light microscope.

To ascertain whether reset traps of both species are able to fire in the air and aspirate in an air bubble, the simple test after Adamec and Poppinga (2016) was conducted. The test is based on the observation that cut off traps of aquatic *Utricularia* species can normally, reliably and repeatedly fire (either spontaneously or after a mechanical stimulation) in moist air after a resetting period of several hours as the water pumping mechanism runs also in moist air. Twenty freshly cut off traps of each species 1–1.8 mm long without air bubble were placed on two layers of filter paper wetted slightly with a diluted mineral solution in a 6 cm plastic Petri dish. The Petri dishes were exposed in a low-volume growth chamber at 25 ± 1°C in fluorescent light for 24 h. After the exposure, traps with a spontaneously aspirated air bubble were counted using a loupe with 5 times magnification. The remaining traps were gently stimulated to fire by a fine brush (like Płachno et al., 2015). However, due to the specific shape and coloration of traps of both species, air bubbles were poorly visible. To test whether *U. westonii* traps are hermetically closed, 14 excised traps 1.2–2 mm long were impaled at their bases by a fine glass capillary of the diameter of ca. 120 μm similar to Adamec and Poppinga (2016). The capillary was connected with a fine syringe (1 ml) and the impaled trap was immersed ca. 5 mm under water. Air was gently pushed into the trap and the place where the air escaped from the trap was observed using the loupe.

## Results

### *Utricularia westonii* (Figure 1A)

**Figure 1:**
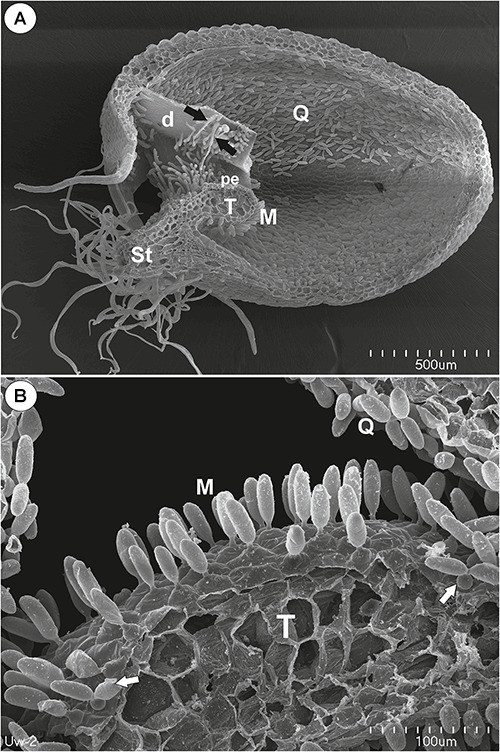
The general structure of the trap of *Utricularia westonii*. **(A)** SEM image of a longitudinal section of an *Utricularia westonii* trap: stalk (St), door (d), door tripping bristles (arrows), the threshold (T), pavement epithelium (pe), monofids (M), quadrifids (Q); scale bar = 500 μm. **(B)** SEM image of a threshold (T) with monofids (M), bifids (arrow) and trap walls with quadrifids (Q); scale bar = 100 μm.

#### Threshold Trichomes

**Figure 2:**
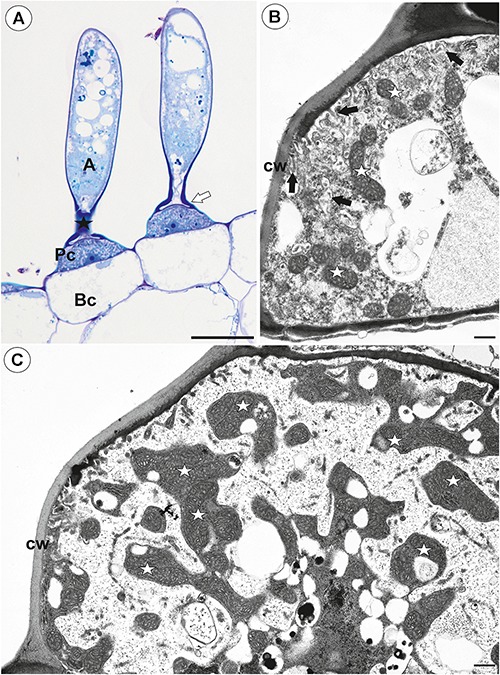
Structure of one-arm trichomes (monofids) from the threshold of *Utricularia westonii* trap. **(A)** A semi-thin longitudinal section of the monofids showing the general structure of trichomes: basal cell (Bc), pedestal cell (Pc), “foot” of terminal cell (white arrow), stalk of terminal cell (black star), arm of terminal cell (A); scale bar = 20 μm. **(B)** The ultrastructure of the monofid pedestal cell; lateral cell wall (cw) impregnated by cutin, cell-wall ingrowths (black arrow), mitochondrion (white star); scale bar = 0.7 μm. **(C)** The ultrastructure of the monofid pedestal cell, note that the most of the cell volume is filled by cell-wall ingrowths and numerous mitochondria (white star) in cytoplasmic pockets; lateral cell wall (cw) impregnated by cutin; scale bar = 0.65 μm.

On the underside of the threshold, there were two types of trichomes: monofids (one-armed trichomes) and bifids (two-armed trichomes) (Figure 1B). In the examined traps, monofids prevailed, while bifids appeared at the lateral parts of the threshold (Figure 1B). The monofids mostly lay adjacent to each other forming the compact cluster of trichomes. Each monofid consisted of a single basal cell, single pedestal cell and a single terminal cell. The terminal cell had a complex structure and consisted of a small “foot” (which lay on the pedestal cell), a stalk and an arm (Figure 2A). The pedestal cell had a circular, broadly subconical base. The lateral wall of the pedestal cell was impregnated by cutin (Figure 2B,C) so this cell acts as a barrier cell. At the distal transverse cell wall, wall ingrowths were formed (Figure 2B,C). Wall ingrowths occurred also on the lateral walls, except where the walls were completely impregnated (Figure 2B,C). The cell-wall ingrowths filled a large part of the pedestal cell. Thus, the pedestal cell was a transfer cell. However, the level of differentiation of the cell-wall ingrowths depended on the activity of trichome cells. Numerous mitochondria occurred in cytoplasmic pockets between the cell-wall ingrowths (Figure 2C). The nucleus and cytoplasm of the arm was concentrated toward its base where it joined the stalk. Cell walls of the arm had small ingrowths. Bifids had similar structure like those in other *Utricularia* species.

**Figure 3:**
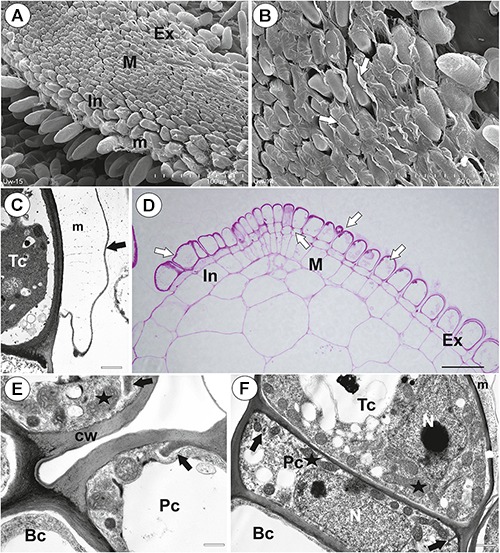
Structure of the pavement epithelium of *Utricularia westonii* trap. **(A)** SEM image of a pavement epithelium showing the three zones of the pavement epithelium (Ex, external; M, middle; In, internal); mucilage (m); scale bar = 100 μm. **(B)** SEM image of a pavement epithelium showing exfoliated cuticles of terminal cells of the external trichomes of pavement epithelium; cuticles which formed velum (arrows); scale bar = 50 μm. **(C)** A trichome of the external zone of pavement epithelium; note mucilage (m) and ruptured cuticle (black arrow); terminal cell (Tc); scale bar = 1.7 μm. **(D)** A semi-thin longitudinal section of the pavement epithelium, showing the three zones of the pavement epithelium (Ex, external; M, middle; In, internal); PAS reaction, note the intensive pink coloration in places where cell-wall ingrowths occurred (white arrows); scale bar = 20 μm. **(E)** Ultrastructure of two trichomes of the external zone pavement epithelium, note cell-wall ingrowths (black arrows) and lateral cell wall impregnated by cutin (cw); basal cell (Bc), pedestal cell (Pc), mitochondrion (black star), scale bar = 0.6 μm. **(F)** Ultrastructure of the internal zone pavement epithelium of a trichome; basal cell (Bc), pedestal cell (Pc), terminal cell (Tc), nucleus (N), cell-wall ingrowths (black arrows), mitochondrion (black star), mucilage (m); scale bar = 1 μm.

The pavement epithelium consisted of glandular trichomes (Figure 3A–F). There were three distinct zones in the pavement epithelium (Figure 3A,D): an external zone, a middle zone and an internal zone that had mucilage trichomes. The trichomes of the external zone had a prominent ruptured cuticle in the terminal cells (Figure 3B,C). These trichomes produced mucilage which was well visible in TEM (Figure 3C). After the PAS reaction, the cell-wall ingrowths were visible in cells of pavement epithelium trichomes, especially in the middle and internal zone (Figure 3D). Cell-wall ingrowths occurred in pedestal (Figure 3F) and terminal cells. Cell-wall ingrowths occurred also in pedestal cells of pavement epithelium trichomes of the external zone (Figure 3E). The trichomes of the internal zone produced mucilage, which was well visible in the SEM and TEM (Figure 3A,F). In all pavement epithelium trichomes, the lateral wall of the pedestal cells was impregnated by cutin (Figure 3E,F).

#### Internal Trichomes

**Figure 4:**
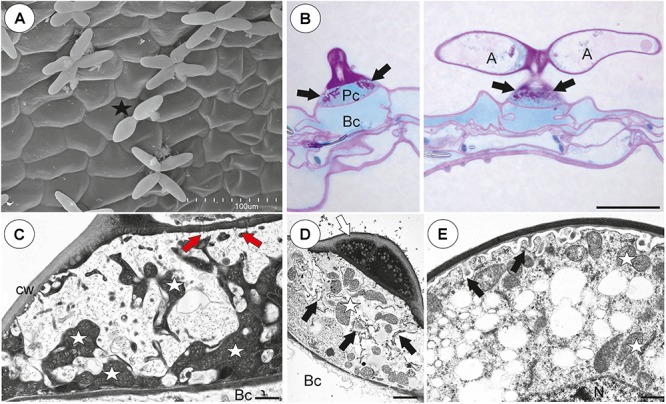
Structure of internal trichomes of *Utricularia westonii* trap. **(A)** SEM image showing quadrifids and a bifid (black star); scale bar = 100 μm. **(B)** Structure of quadrifids using PAS reaction, note the intensive pink coloration in places where cell-wall ingrowths occurred (black arrows); basal cell (Bc), pedestal cell (Pc), arm of terminal cell (A); scale bar = 20 μm. **(C, D)** Ultrastructure of a pedestal cell of a quadrifid, note that the most of the cell volume is filled by cell-wall ingrowths (black arrows) and numerous mitochondria (white star) in cytoplasmic pockets; lateral cell wall (cw) impregnated by cutin; basal cell (Bc), cell wall of feet of terminal cells (white arrow) impregnated by cutin, plasmodesmata (red arrows); scale bar = 0.85 μm for **C**, scale bar = 2 μm for **D**. **(E)** Part of an arm of a terminal cell of a quadrifid; cell-wall ingrowths (black arrows), mitochondrion (white star), nucleus (N); scale bar = 0.8 μm.

On the inner lateral trap walls, there were mainly four-armed trichomes (quadrifids); however, trifids (three-armed trichomes) and bifids also rarely occurred (Figure 4A). Quadrifids had a typical structure: a basal cell, a pedestal cell and four terminal cells (Figure 4A,B). After the PAS reaction, the cell-wall ingrowths were visible in the pedestal cell of quadrifids (Figure 4B). The cell-wall ingrowths arose from the distal transverse wall and a part of the outer lateral wall (Figure 4C,D). Numerous mitochondria occurred in cytoplasmic pockets between cell-wall ingrowths (Figure 4C,D). The lateral wall of the pedestal cell was impregnated by cutin (Figure 4C). The cell wall of the “foot” (which lay on the pedestal cell) and the stalk of terminal cells was thick and partially impregnated by cutin (Figure 4D). The cell wall of the arm had small ingrowths (Figure 4E).

#### External Glandular Trichomes

**Figure 5:**
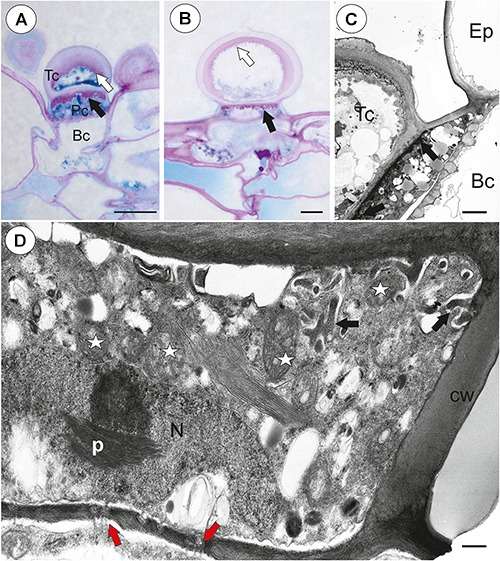
Structure of external glandular trichomes of *Utricularia westonii* trap. **(A, B)** A semi-thin longitudinal sections of trichomes, PAS reaction, note the intensive pink coloration in places where cell-wall ingrowths occurred (black arrows); basal cell (Bc), pedestal cell (Pc), terminal cell (Tc), thick external cell wall of the terminal cell with a PAS positive layer (white arrow); scale bar = 10 μm for **A** and = 5 μm for **B**. **(C)** Ultrastructure of trichome; cell wall ingrowths in pedestal cell (black arrows), terminal cell (Tc), basal cell (Bc), epidermal cell (Ep); scale bar = 1.8 μm. **(D)** Section through a pedestal cell of an external trichome showing cell wall ingrowths (black arrows), mitochondria (star), impregnated by cutin lateral wall of the pedestal cell (cw), nucleus (N), para-crystalline inclusions in the nucleus (p), plasmodesmata (red arrows).

Each external glandular trichome consisted of a basal epidermal cell, a squatting pedestal cell and a single terminal cell (Figure 5A,B). In the pedestal cell, there were cell-wall ingrowths on the outer transverse wall (Figure 5A–D). After the PAS reaction, the cell-wall ingrowths were well visible (Figure 5A,B). The lateral wall of the pedestal cell was impregnated by cutin (Figure 5D). The terminal cell had a thick outer cell wall, which consisted of several layers (Figure 5C). The innermost layer showed a strong reaction for polysaccharide (PAS reaction, Figure 5A,B). The outermost layer is cuticularised (Figure 5C).

#### Trap Vascularization

The vascular bundle (phloem + xylem) traversed the stalk into the trap body, later run along the ventral and later dorsal side of the trap and continued up to the upper part of the entrance.

### *Utricularia multifida* (Figure 6A)

**Figure 6:**
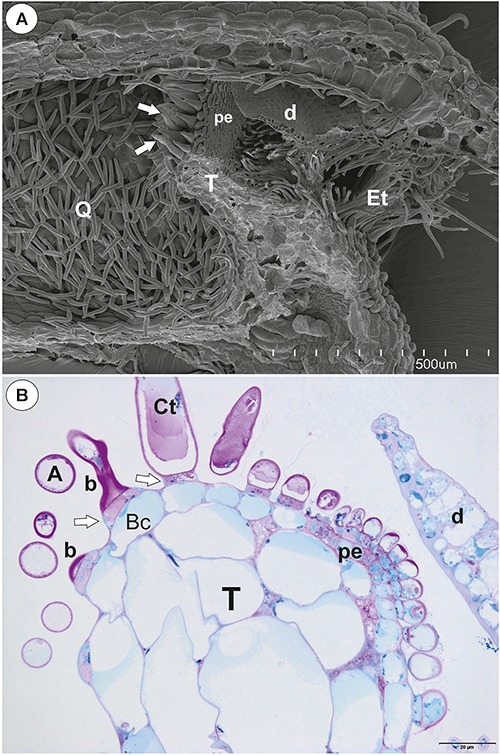
The general trap structure of *Utricularia multifida*. **(A)** SEM image of a longitudinal section of a trap: door (d), conical trichomes (white arrows), threshold (T), entrance trichomes (Et), pavement epithelium (pe), quadrifids (Q); scale bar = 500 μm. **(B)** A semi-thin longitudinal section of threshold, PAS reaction, showing bifids (b), conical trichomes (Ct), pavement epithelium (pe); arm (A), pedestal cell (white arrow), door (d); scale bar = 20 μm.

#### Threshold Trichomes

**Figure 7:**
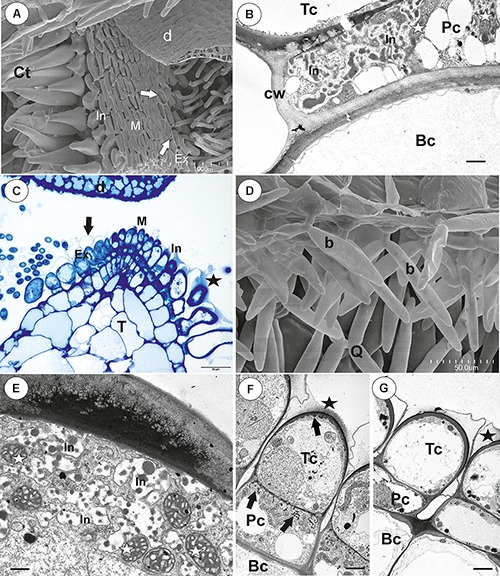
Threshold trichomes of *Utricularia multifida* trap. **(A, B)** SEM image of trap entrance showing the three zones of the pavement epithelium (Ex, external; M, middle; In, internal) and a honeycomb-structure velum (white arrow), conical trichomes (Ct), trap door (d); scale bar = 100 μm. A part of longitudinal section of a conical trichome; cell-wall ingrowths (In), basal cell (Bc), pedestal cell (Pc), terminal cell (Tc), lateral cell wall (cw) impregnated by cutin, mitochondrion (m); scale bar = 0.75 μm. **(C)** A semi-thin nearly longitudinal section of threshold (T) showing the three zones of the pavement epithelium (Ex, external; M, middle; In, internal) and velum (black arrow), conical trichomes. Note mucilage (black star) which covers the pavement epithelium trichomes and conical trichomes; scale bar = 20 μm. **(D)** Cluster of bifids (b), note also quadrifids (Q) on the trap wall; bar = 50 μm. **(E)** Ultrastructure of a pedestal cell of a bifid, note cell-wall ingrowths (In) and mitochondria (white star); scale bar = 0.4 μm. **(F, G)** Structure of pavement epithelium trichomes; wall ingrowths (black arrows), basal cell (Bc), pedestal cell (Pc), terminal cell (Tc), mucilage (black star); scale bar = 1.8 μm for **F** and **G**.

There was continuity between pavement epithelium and glandular trichomes of the inner part of the threshold (Figure 6A,B). At the underside of the threshold, there were three types of trichomes (Figure 6A,B, 7A–D): trichomes with large conical cells (conical trichomes, two or three rows of these trichomes Figure 6B, 7A), monofids (rare) and bifids (Figure 6B, 7D). In the examined traps, conical trichomes and bifids prevailed. Each conical trichome consisted of a single basal cell, a single discoid pedestal cell and a single conical terminal cell (Figure 6B, 7B). Lateral and transverse walls of the pedestal cell were impregnated by cutin (Figure 7B). In the pedestal cell, the cell-wall ingrowths arose from the distal transverse wall (Figure 7B). Small cell-wall ingrowths occurred in the terminal cell, so this cell was a transfer cell. Terminal cells had an exfoliated cuticle. In some traps, these trichomes were covered by mucilage (Figure 7C). Both monofids and bifids had a typical structure. Pedestal cells of these trichomes were barrier and transfer cells (Figure 7E).

The pavement epithelium consisted of glandular trichomes (Figure 6A, 7C), which consisted each of three types of cells: basal, pedestal and terminal (Figure 7F,G). There were three distinct zones in the pavement epithelium (external, middle, internal; Figure 7A). The external-zone trichomes had terminal cells with interrupted cuticles (forming velum; Figure 7A) so that cell walls and mucilage were visible (Figure 7G). The internal-zone trichomes had terminal cells with an exfoliated cuticle and these trichomes produced mucilage (Figure 7C). Cell-wall ingrowths occurred in pedestal and terminal cells of trichomes especially in the middle and internal zone (Figure 7F).

#### Internal Trichomes

**Figure 8:**
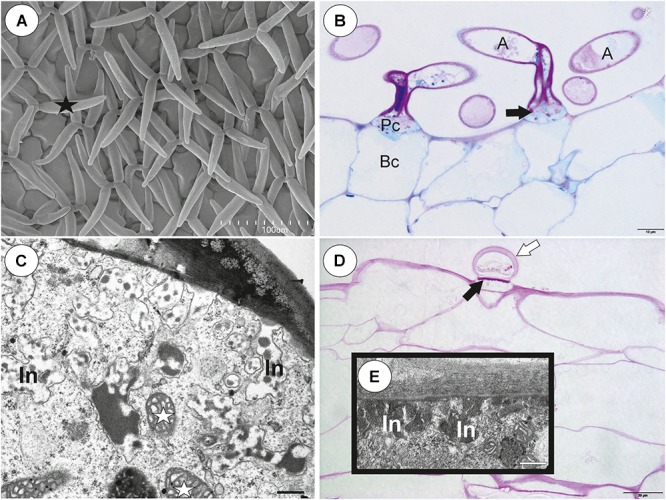
Structure of internal and external trichomes of *Utricularia multifida* trap. **(A)** SEM image showing quadrifids and trifid (black star); scale bar = 100 μm. **(B)** Structure of quadrifids using PAS reaction, note the pink coloration in places where cell-wall ingrowths occurred in pedestal cell (black arrows); basal cell (Bc), pedestal cell (Pc), arm of terminal cell (A); scale bar = 10 μm. **(C)** Ultrastructure of a pedestal cell of a quadrifid, note cell-wall ingrowths (In) and mitochondria (white star); scale bar = 0.5 μm. **(D)** A semi-thin longitudinal sections of an external trichome, PAS reaction, note the intensive pink coloration in places where cell-wall ingrowths occurred (black arrows); thick external cell wall of a terminal cell (white arrow); scale bar = 20 μm. **(E)** A part of section through a pedestal cell of an external trichome showing cell-wall ingrowths (In); scale bar = 0.5 μm.

On the inner lateral trap wall surface, there were mainly four-armed trichomes (quadrifids), however, trifids (three-armed trichomes) also rarely occurred (Figure 8A). Quadrifids had a typical structure: a basal cell, a pedestal cell and four terminal cells (Figure 8B). The pedestal cell had a character of barrier and transfer cell (Figure 8C).

#### External Glandular Trichomes

Each external glandular trichome consisted of a basal epidermal cell, a squatting pedestal cell and a single terminal cell (Figure 8D). The lateral wall of the pedestal cell was impregnated by cutin. In pedestal cells, the cell-wall ingrowths arose from the distal transverse wall (Figure 8E) and were well visible after the PAS reaction (Figure 8D). The terminal cell had a thick outer cell wall (Figure 8D), which consisted of several layers.

#### Trap Vascularization

The vascular bundle (phloem + xylem) traversed the stalk into the trap body, and later runs along the ventral and later dorsal side of the trap and continued up to the upper part of the entrance.

#### Biophysical Experiments

In *Utricularia multifida*, 15% of traps spontaneously had a small air bubble inside after the 24 h exposure but none of them were able to aspirate in any air bubbles after mechanical stimulation (Table 1). In *U. westonii*, 60% traps spontaneously had an air bubble, but only 25% of the remaining traps (2 out of 8) were able to gain an air bubble after mechanical stimulation. In 6 out of the total impaled 14 traps of *U. westonii*, the gently pushed air escaped through the door, while it escaped through the impalement point in the other traps.

**Table 1:** Results of the preliminary test on excised traps of *U. multifida* and *U. westonii* to aspirate in an air bubble both spontaneously during a 24 h exposure and after a mechanical stimulation of the remaining traps in a moist chamber. For each species, the initial trap number was 20.

Species	No. of traps with spontaneous aspiration of air bubble after 24 h	No. of the remaining traps which aspired in air bubble after mechanical stimulation
*U. multifida*	3	0 (out of 17)
*U. westonii*	12	2 (out of 8)

## Discussion

According to Sasago and Sibaoka (1985a,b), in *Utricularia* traps, bifids take part in water pumping so that water is exuded from the pavement epithelium close to the door. Fineran’s team (Fineran and Lee, 1975, 1980; Fineran, 1980, 1985; Fineran and Gilbertson, 1980) also proposed this; however, they added that also quadrifids take part in water pumping and external trichomes excrete water from the trap. If *U. multifida* and *U. westonii* actively generate negative pressure in their traps, they should have well-developed structures such as external trap glands, quadrifids, internal glands near the entrance (bifids) and also those of the pavement epithelium – structures which are all considered to be involved in the water outflow from *Utricularia* traps.

Both *Utricularia multifida* and *U. westonii* traps, as we present here, had structures with mature external glandular trichomes (glands) very similar to those described in *Utricularia monanthos* (=*U. dichotoma*) traps by Fineran and Lee (1980; see also Fineran, 1985, 1980). These authors, based on ultrastructural characters, proposed that mature external glands of *Utricularia* traps are responsible for secreting water. Because the same characters (e.g., pedestal cell with cutinized lateral cell walls and cell-wall ingrowths, thick outer cell wall of terminal cell) occurred in trichomes of *U. multifida* and *U. westonii*, we propose a similar function for their traps.

In the case of the quadrifid structures, both *U. multifida* and *U. westonii* do not differ from other *Utricularia* species (Fineran and Lee, 1974, 1975; Vintéjoux, 1974; Fineran, 1985; Heide-Jørgensen, 1989; Płachno and Jankun, 2004; Vintéjoux and Shoar-Ghafari, 2005). The architecture of these trichomes are the same, and both terminal and pedestal cells are transfer cells. Thus, these characters support the view that these trichomes play a role in nutrient transport. However, the presence of cell-wall ingrowths may not be associated only with water transport, because quadrifids play roles mainly in the secretion of digestive enzymes and the absorption of digested products (Fineran and Lee, 1975; Fineran and Gilbertson, 1980; Fineran, 1985; Heide-Jørgensen, 1989; Płachno et al., 2006; Płachno and Muravnik, 2018). The occurrence of rare trifids and bifids among the quadrifids testifies that there are some disorders in mitotic divisions of terminal cells.

Taylor (1989) noted bifids, quadrifids and rarely trifids but no monofids in *Utricularia westonii* traps. Because monofids prevailed over bifids in *U. westonii* traps examined by us, we suggest that there is probably some trap dimorphism in this species similar to that described in *U. multifida* by Lloyd (1936, 1942). Interestingly, monofids were found in species unrelated to *U. multifida* and *U. westonii* from the subgenus *Bivalvaria* (e.g., sect. *Benjaminia*, sect. *Stomoisia*, sect. *Nigrescentes*, sect. *Oligocista*) and the subgenus *Utricularia* (sect. *Stylotheca*, sect. *Utricularia*; *U. benjaminiana*) (Taylor, 1989; Yang et al., 2009). Yang et al. (2009) proposed that in the genus *Utricularia*, there is an evolutionary trend of reduction in the arm number of trap trichomes. Thus, it is possible that the presence of monofids could represent apomorphies in the *Utricularia* genus. In addition to the terminal cell numbers of the threshold trichomes, we showed that both conical trichomes, monofids and bifids have pedestal cells which played a function as both a barrier and transfer cell, so all these types of trichomes may play a role in water absorption and pumping.

In *Utricularia* traps, a part of the pumped water is probably expelled from the cells of the trichomes of pavement epithelium (Sasago and Sibaoka, 1985a; Fineran, 1985; Adamec, 2018). This is associated with the occurrence of transfer cells in these trichomes (Broussaud and Vintéjoux, 1982; Fineran, 1985; Heide-Jørgensen, 1989; Płachno et al., 2005). In both *U. multifida* and *U. westonii*, we also found these cells in the trichomes of pavement epithelium.

Lloyd (1936, 1942) found velum in the traps of *U. multifida*, however, Westermeier et al. (2017) have recently questioned the occurrence of velum in the traps of this species. The lack of velum may suggest that the *U. multifida* trap functions passively. However, we recorded distinct velum in traps of both *U. multifida* and *U. westonii*. Płachno et al. (2019) proposed two main types of velum: velum with the balloon-like structure (e.g., in sect. *Utricularia* and *Orchidioides*) and the honeycomb-like velum (members of subg. *Polypompholyx*, some *Utricularia* species of subg. *Bivalvaria*). Velum of *U. multifida* and *U. westonii* resembles the latter type.

Lang (1901) noted a well-developed vascular bundle in *U. multifida* traps. We recorded a vascular bundle (contained both phloem and xylem elements) in traps of both *U. multifida* and *U. westonii*. The pattern of trap vascular bundles in *U. multifida* and *U. westonii* was similar to that reported for other species from the subgenera *Polypompholyx* and *Bivalvaria* (Płachno et al., 2017). It cannot be ruled out that some proportion of water may be transported from the traps by xylem to emergent parts of plants to cover transpiration losses.

Generally, the results of the preliminary biophysical investigations on *Utricularia multifida* and *U. westonii* traps are partly ambiguous. The inability of reset *U. multifida* traps to aspirate in an air bubble after even strong and repeated mechanical stimulation suggests that there is no negative pressure inside the trap and thus, firing is excluded. However, as there are many trichomes at the entrance and the door is relatively small and narrow, these characters may interfere with aspiration of air bubbles as well as with mechanical stimulation.

The fact that 60% of the *U. westonii* traps were able to aspirate in an air bubble in 24 h, but unable to fire after a strong stimulation, might indicate that the air entered the traps due to drought in the moist Petri dish rather than due to spontaneous firing. Moreover, the ability to easily push air through the *U. westonii* door (entrance) from the inner trap side might indicate that the trap is permanently open and/or the door possesses a very low mechanical stiffness. Therefore, it is possible to suggest that traps in both species function passively like an eel trap, however, other explanations should also be considered. E.g., traps were taken from stressed plants (taken from *in-vitro* culture to *ex-vitro* conditions; these plants rotted and died several weeks after the experiment). Westermeier et al. (2017) noted that aquatic species are generally much easier to trigger manually than terrestrial ones. Reifenrath et al. (2006) had problems to prove the trigger-hair function in several terrestrial species (*U. sandersonii*, *U. prehensilis*, *U. calycifida*, *U. longifolia*, *U. subulata*), but did not propose non-suction mechanism for their traps. Significantly, Lloyd (1942) showed that a suction mechanism occurred in traps of *U. multifida*. We propose that trap physiology of species of subg. *Polypompholyx* could be different from that of subg. *Utricularia*. Thicker and more stiff trap walls as well as a well-developed vascular bundle with tracheary xylem elements should be considered for the trap functioning of subg. *Polypompholyx*. For example, there is a distinct lag-period after trap firing in two accessions of aquatic *U. dichotoma* (Płachno et al., 2015). Thus, this suggests that water-pumping regulation varies among subgenera or even among sections in the *Utricularia* genus. Westermeier et al. (2017) noted that *U. menziesii* spontaneously fired once, so trap physiology of this species may be different. In this vein, similar *U. multifida* and *U. westonii* traps might also have different firing behavior. It is also possible that trap functioning of *U. multifida* and *U. westonii* is associated with trap dimorphism. Small traps might behave differently than large ones. Westermeier et al. (2017) noted that in some species (*U. calycifida*, *U. dichotoma, U. longifolia*, *U. welwitschii*), there was a different firing behavior among traps even on the same stolon.

The species from the subg. *Polypompholyx* have intrigued because of their four-lobed calyx and also their trap morphology. The hypothetical “passive” trap mechanism was also applied as supporting a possible close relationship between the funnel-like *Genlisea* mechanism (without any suction of water; see Adamec, 2003) and the primitive conditions of the subg. *Polypompholyx* traps (Taylor, 1989). Nonetheless, this interpretation assuming its “basal” position (Reifenrath et al., 2006) has led to the erroneous idea (Crisp and Cook, 2005; Zachos, 2016) of *Polypompholyx* species as a possible link between the passive traps of *Genlisea* (Adamec, 2003) and the active ones of *Utricularia*. However, only a few *Genlisea* species have experimentally been examined for the occurrence of water flow in the traps so far (Adamec, 2003; Płachno et al., 2008). Thus, the problem of *Genlisea* trap functioning has not been studied enough to make sure the system is entirely passive in the whole *Genlisea* genus.

According to the phylogenetic hypothesis, subg. *Polypompholyx* is monophyletic and is nested as a sister group to the subg. *Bivalvaria* + subg. *Utricularia* clade (the rest of *Utricularia* species) (Reut and Jobson, 2010; Silva et al., 2018). Considering the chronological estimates based on DNA, the last common ancestor of *Utricularia* arose ∼30 (Silva et al., 2018) or 31 mya (Ibarra-Laclette et al., 2013). While the split between the sections *Polypompholyx* and *Pleiochasia* occurred ∼15 mya (Ibarra-Laclette et al., 2013), the common ancestor of *U. westonii* + *U.tenella* + *U.multifida* clade occurred 4.87 mya (Jobson et al., 2017), thus being much more recent than the last common ancestor of the *Utricularia* genus which possibly bore active traps.

## Conclusion

In both investigated species, there is a clear controversion between our anatomical data, showing a normal pressure-based, active trap functioning like in all other *Utricularia* sections, and our simple preliminary experimental data, indicating that the traps are permanently open and, thus, passive. Exact biophysical measurements (*sensu* Adamec and Poppinga, 2016) are needed to answer this question.

## Data Availability

All datasets generated for this study are included in the manuscript and/or the supplementary files.

## Author Contributions

All the authors BP, PŚ, LA, SC, and VFOM discussed the results and commented, corrected, and approved the manuscript.

## Conflict of Interest Statement

The authors declare that the research was conducted in the absence of any commercial or financial relationships that could be construed as a potential conflict of interest.

## Footnotes

1 http://www.bestcarnivorousplants.net/
